# Double-Layered Pebax^®^ 3533/ZIF-8 Membranes with Single-Walled Carbon Nanotube Buckypapers as Support for Gas Separation

**DOI:** 10.3390/membranes13010071

**Published:** 2023-01-06

**Authors:** Víctor Berned-Samatán, Carlos Téllez, Joaquín Coronas

**Affiliations:** 1 Instituto de Nanociencia y Materiales de Aragón (INMA), CSIC-Universidad de Zaragoza, 50018 Zaragoza, Spain; 2 Chemical and Environmental Engineering Department, Universidad de Zaragoza, 50018 Zaragoza, Spain

**Keywords:** single-walled carbon nanotube, buckypaper, metal–organic framework, ZIF-8, gas separation, CO_2_ capture

## Abstract

Single-walled carbon nanotube buckypapers (SWCNT-bps) coated with a metal–organic framework ZIF-8 layer were used as supports for the preparation of Pebax^®^ 3533 TFC membranes by both phase inversion and spin coating techniques. Upon proper characterization of the materials by X-ray diffraction, IR spectroscopy, thermogravimetry and electron microscopy, the obtained membranes were tested in gas separation experiments with a 15:85 CO_2_/N_2_ mixture. These experiments proved that the ZIF-8 layer prevented from the penetration of the polymer selective film into the SWCNT-bp support, giving rise to a highly permeable selective membrane. The optimum membrane was achieved by the spin-coating method, with better permeation results than that prepared by the phase inversion method, obtaining a CO_2_ permeance of 566 GPU together with a CO_2_/N_2_ selectivity of 20.9.

## 1. Introduction

In recent times, climate change and its effects on the planet are more prominent (an increase in temperatures, the retreat of glaciers and extreme weather conditions) [1], which is mainly caused by greenhouse gases. Carbon dioxide (CO_2_) is the most common of these gases, considered the main cause of the increase in global temperature. For that reason, technologies for its capture and sequestration are in high demand [2]. Post-combustion CO_2_ capture is the simplest approach to this problem and could be easily adapted to industrial facilities. Gas separation with membranes is drawing more interest over traditional techniques (absorption with amines, cryogenic distillation and adsorption) [3] due to the relevant properties of membranes such as their mechanical simplicity, lower energy consumption and the low-cost of fabrication and maintenance [4]. 

Polymer membranes exhibit excellent properties and transport parameters for the separation of the CO_2_/N_2_ post-combustion mixture, where the CO_2_ concentration varies between 15% (from the exhaust of a typical combustion process) to 30% (in the case of cement and steel industries). Among the quasi-infinite variety of polymers, poly(ether-block-amide) copolymers (Pebax^®^) are very promising due to the characteristics that their variable composition, based on different proportions of polyamide (PA) and polyether (PE) blocks, confer them [5,6].

An ideal membrane for gas separation should be composed of a selective skin layer exceeding the Robeson trade-off relation between selectivity and permeability [7], as thin as possible with the objective of achieving high permeance (permeability divided by membrane thickness) but mechanically robust [8,9].

A thin-film composite (TFC) membrane consists of a selective layer with very thin thickness, since the mechanical stability relies on the support, which has to be robust, chemically and thermally stable and very permeable. In these conditions, the objective is to achieve a highly permeable TFC membrane with the same separation selectivity observed when the selective membrane polymer material is available as a dense membrane. The selective layer in the TFC membrane can be further improved with the addition of nanoparticles acting as nanofillers, giving rise to the so-called thin-film nanocomposite (TFN) membrane. Among the variety of nanofillers, MOFs (metal–organic frameworks) and special ZIFs (zeolitic imidazolate frameworks), with a generally narrower porosity than typical carboxylate-type MOFs, are widely studied due to their affinity with the polymer matrix [10,11]. ZIF-8 is probably the most applied MOF as a membrane material. In addition, ZIFs are a subfamily of MOFs firstly reported and named by Park et al. [12] that have crystalline topologies similar to those of zeolites. In particular, ZIF-8, under a different name, was first synthesized by Huang et al. [13] consisting of Zn^2+^ ions coordinated to four nitrogen atoms present in the positions 1 and 3 of the 2-methylimidazolate ligand, forming a SOD-type topology present in several other ZIFs with slightly different composition [14].

Another strategy that has been used to improve gas separation in TFC membranes is to incorporate an intermediate layer between the selective layer and the support; this layer sometimes performs the function of a gutter layer [15]. For this function, several nanostructured materials have been used: MOF nanosheets for Polyactive^®^ [16] or polyamide [17] selective layers and COF for Pebax^®^ 1657 selective layers [18], among others.

Carbon nanotubes are promising materials for gas and water filtration based on membranes due to their high specific porosity and surface area, hydrophobicity, mechanical strength and potential functionalization [9,19,20,21]. In addition, they are commercially available. In this work, we report the preparation of Pebax^®^ 3533 TFC membranes with single-walled carbon nanotubes conforming to a buckypaper (SWCNT-bp) as support, which provides a highly permeable and mechanically robust base for obtaining a high-performance membrane. Pebax^®^ 3533 is a suitable polymer for CO_2_ separation due to its composition comprising 75 wt % poly(tetramethylene oxide, PTMO) and 25 wt % aliphatic polyamide (PA12). In addition, a ZIF-8 layer was grown on the SWCNT-bp to avoid the penetration of the Pebax^®^ 3533 into the support porosity, having a gutter layer effect [22,23]. All this configures a unique membrane architecture exhibiting high gas separation performance and stability. 

## 2. Experimental Procedure

### 2.1. Materials

Polyether-block-amide Pebax^®^ 3533 SA 01 MED (75 wt % poly(tetramethylene oxide) (PTMO), 25 wt % aliphatic polyamide (PA12)) in the form of pellets was kindly provided by Arkema, France. Single-walled carbon nanotubes (SWCNT, 1.2–2 nm) ≥93% carbon content and non-ionic surfactant Triton™ X-100 were purchased from Sigma-Aldrich. SWCNTs were used as received; additional characterization of them can be found in our previous publication [9]. 2-propanol (IPA, HPLC grade, ≥99.9%) and methanol (HPLC grade, ≥99.9%) were obtained at Panreac Applichem. 1-propanol, 1-butanol and zinc nitrate hexahydrate (reagent grade) were purchased from Scharlab, while 2-methylimidazole (mIm) (99%) was acquired from Acros Organics. Finally, Nylon™ filter with a diameter of 47 mm and 0.22 µm pore size was purchased from Labbox.

### 2.2. Single-Walled Carbon Nanotube Buckypaper (SWCNT-bp) Preparation

By adapting a previously published technique, free-standing SWCNT films were prepared using a vacuum filtering process [9,24,25,26]. Typically, 10 mg of SWCNT was dispersed in 60 mL of distilled water with 1 wt % of Triton X-100 surfactant using an ultrasound probe sonicator (Sonics Materials VC-750–220, 750 W, 20 kHz) for 1 h. The sonicated suspension was then filtered through a porous Nylon™ filter housed in a glass filtration funnel with a diameter of 47 mm and washed with a 1:1 (*v*/*v*) acetone:2-propanol solution [24]. After allowing it to dry at room temperature overnight, it could be peeled off from the filter, obtaining a 15 µm thick, self-standing SWCNT buckypaper, thick enough as to ensure the needed mechanical strength for the gas phase separation characterization.

### 2.3. ZIF-8 Synthesis on SWCNT-bp

The synthesis of ZIF-8 was performed by adapting a technique that was already published [26,27]. In order to keep one of their sides away from the solutions, the free-standing SWCNT-bp was positioned in between a ring and a disc of stainless steel (Figure S1) vertically in 100 mL of a 10 mM zinc nitrate hexahydrate methanolic solution for 30 min. After being rinsed in fresh methanol for 1 min, the SWCNT-bp was then immersed vertically in 100 mL of a 20 mM 2-methylimidazole methanolic solution for 24 h. Then, it was washed again in pure methanol for 1 min. This treatment was repeated twice. The membrane was then detached from the metal rig, cleaned in pure methanol for 10 min and allowed to dry at room temperature overnight. The entire synthesis process was carried out at ambient temperature.

### 2.4. Preparation of Pebax^®^ 3533 Dense Membranes

The membranes were prepared using a modified reported method [28]. Concisely, 0.3 g of the polymer was dissolved in 6.7 g of 3:1 (*v*/*v*) of 1-propanol:1-butanol mixture under reflux for 1 h at 80 °C. Afterwards, the solution at room temperature was cast into a glass Petri dish and dried at 40 °C for 24 h.

### 2.5. Preparation of Pebax^®^ 3533 Supported Membranes by Phase Inversion (PI) 

A polymer solution was prepared dissolving the required amount of Pebax^®^ 3533 pellets in a 3:1 (*v*/*v*) of 1-propanol:1-butanol mixture stirred under reflux at 80 °C for 1 h, obtaining a 1 wt % Pebax^®^ 3533 solution. The method used for the membrane preparation was based on a previous report [29]. First, the support was fixed horizontally with a vacuum pump. Secondly, the support was put in contact with the Pebax^®^ 3533 solution, previously poured in a Petri dish, for about 2 to 3 s. Then, the support was soaked in distilled water (provoking the phase inversion) in another Petri dish for 2 min. After this, the membrane was gently dried with compressed air. This procedure was repeated three times, after which the membrane was dried at 40 °C for 24 h.

### 2.6. Preparation of Pebax^®^ 3533 Supported Membranes by Spin Coating (SC)

First, a 3 wt % Pebax^®^ 3533 solution was obtained following the previous method. Then, the membrane was prepared following a previously reported protocol [30]. Briefly, the support was introduced in a Laurell WS-650MZ-23NPP/A1/AR1 spin coater and fixed with vacuum. Then, 500 µL of the polymer solution was poured on the support, which was spun at 2500 rpm for 20 s. The membrane was posteriorly dried at 40 °C for 24 h.

### 2.7. Membrane Characterization

Fourier transform infrared spectroscopy (FTIR) was performed with a Bruker Vertex 70 FTIR spectrometer equipped with a DTGS detector and a Golden Gate diamond ATR accessory. The spectra were recorded by averaging 40 scans in the wavenumber range of 4000–600 cm^−1^ at a resolution of 4 cm^−1^. Thermogravimetric analyses (TGA) were carried out using a Mettler Toledo TGA/STDA 851e. Small pieces of membranes (~3 mg) placed in 70 µL alumina pans were heated under an airflow (40 cm^3^(STP) min^−1^) from 35 to 700 °C at a heating rate of 10 °C min^−1^. Scanning electron microscopy (SEM) images were collected with a FEI-Inspect F50 microscope at a voltage of 10 kV. The samples were previously coated with Au/Pd under vacuum conditions, and transversal images were obtained by cutting the cooled samples with liquid nitrogen. X-ray diffraction (XRD) measurements were performed with an Empyrean PANalytical diffractometer with a Cu-Kα source (λ = 1.5406 Å). Data were collected in the 2θ range from 5 to 40° at a scanning rate of 0.01°·s^−1^.

### 2.8. Mixed Gas Permeation

Membranes were cut and placed in a module consisting of two stainless steel pieces and a 316LSS macro-porous disk support (Mott Co., Farmington, CT, USA) with a 20 µm nominal pore size. Membranes, 2.12 cm^2^ in area, were sealed inside the module by means of Viton O-rings. To control the temperature of the experiment (in this work, in the 25–50 °C range), the permeation module was placed in a UNE 200 Memmert oven. The gas separation measurements were carried out by feeding the post-combustion gaseous mixture of CO_2_/N_2_ (15/85 cm^3^(STP) min^−1^) to the feed side at an operating pressure of 3 bar to favor CO_2_ permeation. Two mass flow controllers (Alicat Scientific, Tucson, AZ, USA, MC-100CCM-D) controlled the gas flows. The permeate side of the membrane was swept with 50 cm^3^(STP) min^−1^ of He at atmospheric pressure (~1 bar) (Alicat Scientific, MC-100CCM-D). Concentrations of N_2_ and CO_2_ in the permeate stream were analyzed online by an Agilent 990 micro-chromatograph. Permeances were calculated in GPU (Equation (1)) once steady state at the exit stream was reached (after at least 2 h). The separation selectivity (α) (Equation (2)) was calculated as the ratio of the corresponding permeances (P).

1 GPU = 10^−6^ cm^3^(STP) cm^−2^ s^−1^ cm Hg^−1^ (1)

α = *P*_a_/*P*_b_ (2)

## 3. Results and Discussion

### 3.1. Membrane Characterization

**Figure 1:**
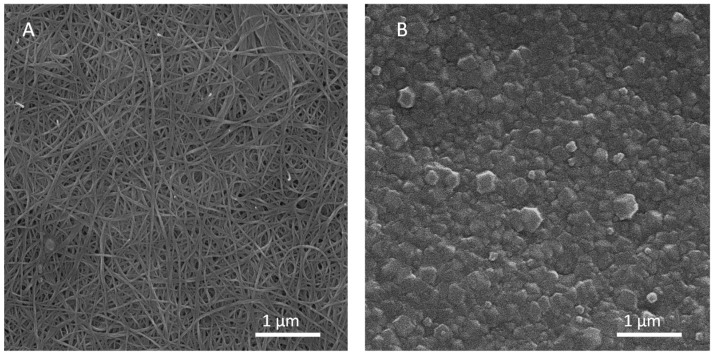
SEM images of the surface of the SWCNT-bp (**A**) and the ZIF-8/SWCNT-bp (**B**).

Figure 1 shows the surface of the SWCNT-bp support before (Figure 1A) and after ZIF-8 synthesis (ZIF-8/SWCNT-bp, Figure 1B). It can be seen the open structure of the interweaved SWCNT-bp, as has been observed previously [26], and that the ZIF-8 layer provides a continuous coating of intergrowth MOF crystals on top of the support with a thickness of ca. 90–100 nm.

On top of the ZIF-8 layer, a selective Pebax^®^ 3533 coating was added via two different methods. Pebax^®^ 3533 was chosen for this purpose for its hydrophobic nature (as compared to other Pebax^®^ codes with a lower content of the PE segment) [29], which is compatible with both SWCNT and ZIF-8. This, along with the solvent used in the polymer solution (1-propanol/1-butanol mixture), make Pebax^®^ 3533 an ideal choice for the membrane preparation.

**Figure 2:**
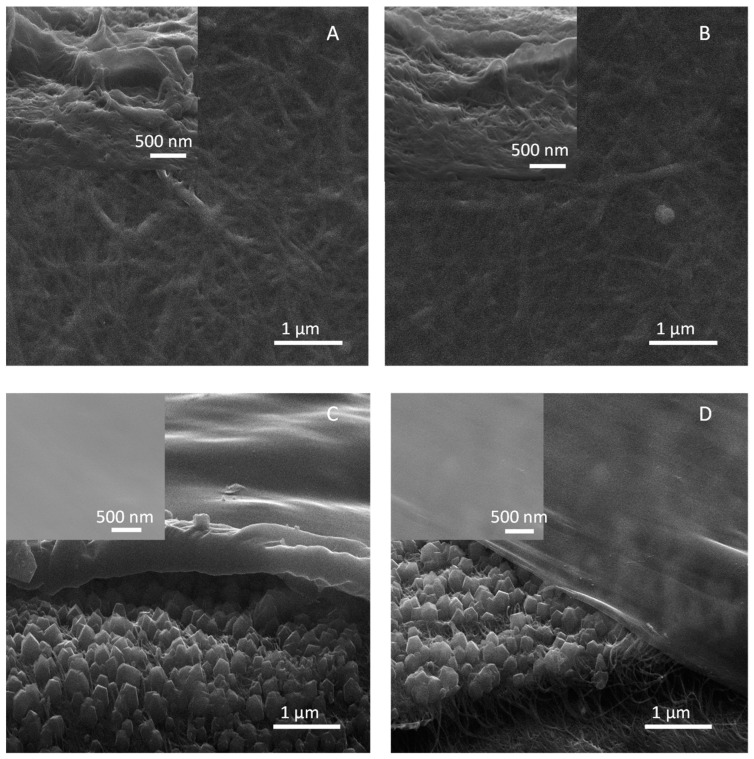
SEM images of the surfaces of Pebax^®^/SWCNT-bp-PI (**A**) and Pebax^®^/SWCNT-bp-SC (**B**). Cross-section images of Pebax^®^/ZIF-8/SWCNT-bp-PI (**C**) and Pebax^®^/ZIF-8/SWCNT-bp-SC (**D**). The insets correspond to the cross-sections of the membranes (**A**,**B**) and their surfaces (**C**,**D**). Cross-section images were obtained by cutting the samples under liquid nitrogen, which causes the apparent gap between the Pebax^®^ and ZIF-8 layers.

The first Pebax^®^ 3533 coating method was phase inversion (PI), carried out by the previously mentioned protocol forming the Pebax^®^/SWCNT-bp-PI (Figure 2A) and Pebax^®^/ZIF-8/SWCNT-bp-PI (Figure 2C) membranes. In addition, a spin-coating (SC) method was applied following the previously mentioned conditions, producing the Pebax^®^/SWCNT-bp-SC (Figure 2B) and Pebax^®^/ZIF-8/SWCNT-bp-SC (Figure 2D) membranes. These two pairs of Pebax^®^ coatings on both bare SWCNT-bp and ZIF-8/SWCNT-bp allow one to infer the importance of the MOF layer. In fact, the SEM images in Figure 2A,B depict that the SWCNT-bp surface is continuously covered with the polymer in the case of the Pebax^®^/SWCNT-bp. However, none of the two applied methods can avoid, as seen in the cross-section images, the penetration of the polymer inside the SWCNT-bp support.

Membranes with the ZIF-8 layer also show a good coating with the polymer without observing the SWCNT-bp support on the surface (insets in Figure 2C,D). In addition, their cross-section images indicate that Pebax^®^ penetration into the support was avoided thanks to the ZIF layer. In these two cases, the thicknesses of the layers can be deduced from Figure 2C,D. The ZIF-8 layer is around 90–100 nm thick. In addition, although this ZIF layer may not be well intergrown, it should be known that the selective layer is going to be, as will be verified in the separation measurements, the polymer layer. The main difference between membranes Pebax^®^/SWCNT-bp-SC and Pebax^®^/ZIF-8/SWCNT-bp-SC resides in the Pebax^®^ thickness achieved with each method. The phase inversion method formed a denser polymer layer with a thickness in the ca. 300–350 nm range, whilst the spin coating method provided a thinner and more regular layer ca. 100–120 nm thick. This will be of paramount importance when evaluating the separation properties of the two membranes.

**Figure 3:**
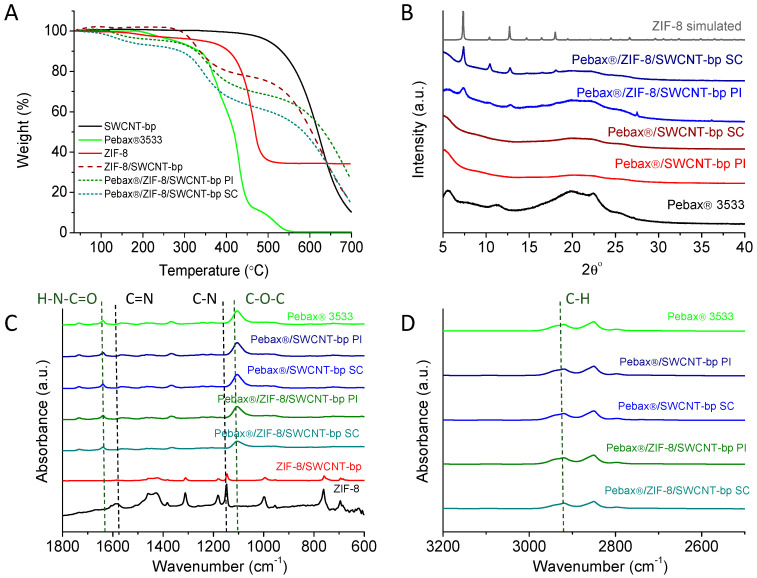
TGA curves (**A**), XRD diffractograms (**B**) and FTIR-ATR spectra (**C**,**D**) of the membranes.

The thermal stability of the membranes was studied by thermogravimetric analysis (TGA). The TGA results are visualized in Figure 3A. It can be seen that ZIF-8 is stable up to 425 °C, which is in agreement with the literature [31]. Nevertheless, the degradation of the ZIF-8 layer begins above 300 °C, which is due to the greater accessibility of oxygen to the thin layer of ZIF-8. Regarding Pebax^®^, it can be observed that its main degradation begins above 350 °C, which is also in agreement with the literature [29]. In multilayer membranes, the degradation of Pebax^®^, given its smaller thickness, is more difficult to discern, but it would take place coupled with that of the ZIF, which would also be related to the good integration of these two materials. However, a greater loss of solvents (weight loss below 200 °C) was observed in the membranes with Pebax^®^. Regarding the TGA of the SWCNTs, their degradation does not start until 550 °C having at 700 °C a remaining weight; this thermal stability has already been observed previously [9]. In the membrane, it can also be seen that an incomplete degradation was observed at 700 °C due to the presence of SWCNTs and ZnO formation from ZIF decomposition.

The XRD diffractogram shown in Figure 3B confirms the correct synthesis of the ZIF-8 layer (with some shielding from the polymer coating) with the presence of the most intense and characteristic peaks of ZIF-8 (7.4, 10.4 and 12.7°) in agreement with the simulated pattern [32]. It can also be seen the characteristic peaks of Pebax^®^ 3533 at 5.8, 11.3 and 22.4° [29,33,34]. In addition, the more defined ZIF-8 peaks in the Pebax^®^/ZIF-8/SWCNT-bp-SC correlate with the lower thickness of the polymer layer obtained by the spin-coating technique and observed by SEM (Figure 2C,D).

Figure 3C,D show the characteristic bands in FTIR-ATR corresponding to ZIF-8 at 1583 cm^−1^ for the C=N bond and at 1149 and 995 cm^−1^ for the C-N bond [35]. Regarding Pebax^®^ 3533, it has characteristic bands at 1639 cm^−1^ (the vibration of the H-N-C=O group), 2935 cm^−1^ (the bending of the C-H aliphatic chain) and 1101 cm^−1^ (the C-O-C ether group) [36,37,38]. In the membrane in which ZIF-8 was synthesized, some of the characteristic peaks of ZIF-8 can be observed, which were mostly preserved when the Pebax layer was deposited. Some of the characteristic peaks of Pebax^®^ 3533 were detectable on the TFC membranes.

### 3.2. Mixture Gas Permeation Tests

**Figure 4:**
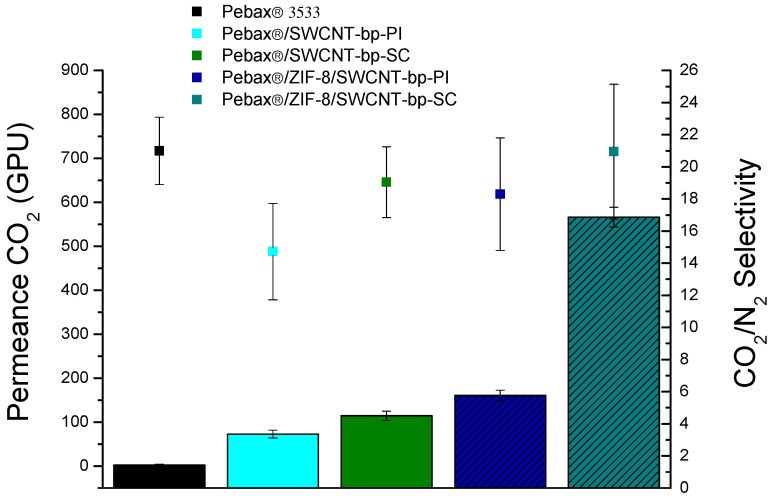
Gas permeance (bars) and selectivity (squares) for the CO_2_/N_2_ (15:85) gas mixture at 35 °C and 3 bar feed pressure for the membranes prepared in this work.

The gas separation performance of the membranes was tested for the 15:85 CO_2_/N_2_ mixture at 35 °C and 3 bar feed pressure. Three different membrane samples of each type were evaluated at the same conditions to demonstrate the reliability of the membranes and their preparation procedures, allowing, in turn, the calculation of the error bars shown along this section. The results of the experiments are depicted in Figure 4, where the parameters corresponding to a Pebax^®^ 3533 dense membrane are also shown for comparison.

All the data shown in Figure 4 are also collected in Table S2. Concerning the separation results for the Pebax^®^/SWCNT-bp membranes, a slight improvement in permeance in relation to the dense membrane but with a decline in the selectivity, less accused in the SC membrane, was obtained. This correlates with the fact that the polymer would have penetrated into the porosity of the SWCNT support. Compared to the membrane containing the ZIF-8 interlayer, as discussed below, this penetration would simultaneously increase the membrane resistance (giving rise to a lower CO_2_ permeance) and decrease its separation selectivity due to the difficulty of obtaining, in such conditions, a defect-free membrane. What is evident is the increase in permeance with respect to the dense membrane, given its lesser thickness, which would give it, despite the slight decrease in selectivity, a certain advantage. 

When a ZIF-8 layer was placed on top of the SWCNT support and then the polymer was coated on the ZIF, a clear improvement in the CO_2_ permeance was accomplished as compared to the dense Pebax^®^ and to the Pebax^®^ directly prepared on the SWCNTs. In the case of the Pebax^®^/ZIF-8/SWCNT-bp-PI (phase inversion) membrane, the improvement in CO_2_ permeance was not as significant as Pebax^®^/ZIF-8/SWCNT-bp-SC (spin-coating), in line with the fact that the phase inversion method generated a thickness three times larger than the spin-coating method. In these conditions, the membrane Pebax^®^/ZIF-8/SWCNT-bp-SC presents the best results in terms of both CO_2_ permeance (566 ± 22 GPU) and CO_2_/N_2_ selectivity (20.9 ± 4.2). In fact, this selectivity value is practically the same as that achieved with the dense bare polymer (21.0), supporting the idea that the polymer skin layer was defect-free. However, CO_2_ permeance increased from 2.4 GPU to 566 GPU, i.e., by a factor of 235. In conclusion, no apparent effect on the selectivity occurs when ZIF-8 is present as an intermediate layer between the SWCNT support and the Pebax^®^ 3533 skin, which is related to a gutter layer effect achieved from the ZIF-8 coating [39,40].

**Figure 5:**
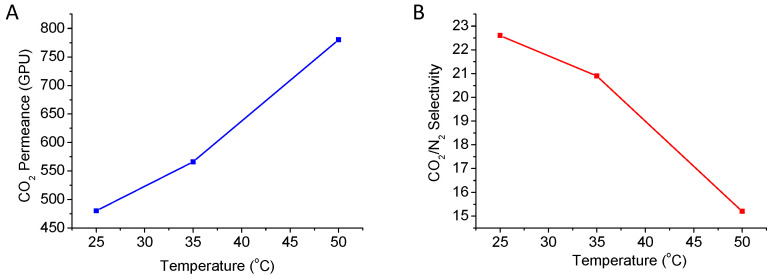
Permeance (**A**) and selectivity (**B**) results for the gas separation experiments carried out at 25, 35 and 50 °C with the Pebax^®^/ZIF-8/SWCNT-bp-SC membrane for CO_2_/N_2_ (15:85) at 3 bar.

In addition, to study permeance and selectivity dependence with temperature, gas permeation tests for the best performing membrane (Pebax^®^/ZIF-8/SWCNT-bp-SC) were carried out between 25 and 50 °C at a 3 bar feed pressure (Figure 5). The Arrhenius model was applied in Figure S2 with Equation (3) [4,41]:

(3) $$P=P_{0}\cdot \exp\left(-\frac{E_{p}}{RT}\right)$$

where *P* is the gas permeance in GPU, *P*_0_ is the pre-exponential factor in GPU, *E_p_* is the permeation apparent activation energy in J·mol^−1^, *R* is the ideal gas constant (8.314 J·mol^−1^·K^−1^) and *T* is the temperature in K.

On the basis of this model, the apparent activation energies for the permeation of CO_2_ and N_2_ are 13.8 kJ·mol^−1^ and 35.1 kJ·mol^−1^, respectively. These results are in concordance with the reported values for Pebax^®^ 3533 membranes, prepared on polymeric supports, of 14.2 kJ·mol^−1^ for CO_2_ and 29.6 kJ·mol^−1^ for N_2_ [29] and justify the reduction in selectivity with the temperature due to the higher activation energy for N_2_ with respect to CO_2_ [42]. It must be taken into account that permeance is proportional to diffusion and solubility, and these two factors affect the defined apparent activation energy. On the one hand, diffusivity, which increases with temperature due to the mobility of molecules and polymer chains, has positive activation energy. On the other, the solubility (adsorption), being an exothermic process, has a negative heat of adsorption. The low value of CO_2_ *E_p_* is due to the fact that adsorption is a predominant factor for this gas, and therefore, the decrease in selectivity is due to the decrease in CO_2_ adsorption. Moreover, these values, together with the fact that the best CO_2_/N_2_ selectivity corresponded to that of the dense membrane, suggest that, beyond the ancillary role of ZIF-8 to properly coat with a thin layer of polymer Pebax^®^ 3533, the contribution of the MOF to the gas transport is not significant, which is in agreement with the fact that the SEM observation did not reveal a perfectly intergrown layer strictly necessary for gas separation.

One of the Pebax^®^/ZIF-8/SWCNT-bp-SC membranes was tested again, after 3 months of room temperature storage with preservation from light, to study the stability of the membrane with time. As collected in Table 1, no appreciable decrease in its separation properties was observed, confirming its stability in terms of CO_2_/N_2_ separation.

**Figure 6:**
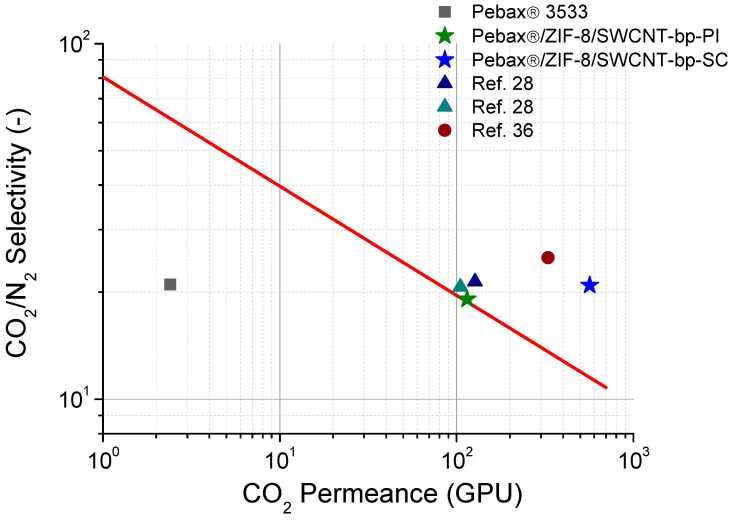
CO_2_/N_2_ selectivity-CO_2_ permeance upper bound defined in GPU at 35 °C (red line). Comparison between TFC membranes based on Pebax^®^ 3533 [29,38] with the membranes obtained in this work, including the dense membrane.

Finally, Figure 6 shows the Robeson-type upper bound for CO_2_/N_2_ with permeance calculated in GPU instead of typical permeability in Barrer. This permits the comparison of the best membranes in this work with other Pebax^®^ TFC membranes observed in the literature, always based on Pebax^®^ 3533. It can be concluded that the membranes developed in this work are amongst the most permeable with high values of CO_2_/N_2_ selectivity.

## 4. Conclusions

It can be concluded that single-walled carbon nanotube buckypapers (SWCNT-bps) can be used as supports for Pebax^®^ 3533 membranes if an intermediate (gutter) layer of MOF ZIF-8 is placed by crystal intergrowth between both materials. Two different methods can be applied for the purpose of preparing the Pebax^®^ thin layer: phase inversion and spin coating. When no ZIF-8 was used and the Pebax^®^ was directly placed on the SWCNT-bp surface, the obtained membranes presented a small increase in CO_2_ permeance values and a decrease in CO_2_/N_2_ selectivity due to the polymer penetration in the support. The ZIF-8 layer placed on top of the SWCNT-bp allowed solving the problem of Pebax^®^ penetration with either of the two polymer coating procedures applied (phase inversion and spin coating). However, the membranes prepared with the spin-coating method, which had less thickness and higher quality, presented the best separation results of this work, with a CO_2_ permeance of 566 ± 22 GPU and a CO_2_/N_2_ selectivity of 20.9 ± 4.2. The permeation testing at different temperatures in the 25–50 °C range allowed estimating the apparent activation energies for both permeating gases. This explains the permeation behavior with the temperature related to the CO_2_ sorption, which is in line with the reported literature, further suggesting that, together with the fact that the best CO_2_/N_2_ selectivity corresponds to that of the dense membrane, the contribution of the MOF to gas transport is not significant. 

Finally, as this current gas separation performance is among the best in the literature for the polymer used, the methodology applied here opens the door for an intermediate ZIF layer, with a function similar to that of a gutter layer, to be used for the development of ultra-thin, high-quality membranes that retain the separation selectivity properties of the dense polymer.

## Figures and Tables

**Table 1:** Permeance and selectivity values of a Pebax^®^/ZIF-8/SWCNT-bp-SC membrane after 90 and 100 cumulative days of storage at room temperature.

Time (days)	CO_2_ Permeance (GPU)	CO_2_/N_2_ Selectivity
0	565	20.5
90	566	20.1
100	564	20.1

## Data Availability

Complementary data available as supporting information and upon request to the authors.

## Supplementary Materials

The following supporting information can be downloaded at: https://www.mdpi.com/article/10.3390/membranes13010071/s1, Figure S1. Photographs of the experimental rig for the synthesis of the ZIF-8 layer on the SWCNT buckypaper (A). The buckypaper placed between the disc and the ring (B). ZIF-8 layer being synthesized on the buckypaper (C) at room temperature; Figure S2. Arrhenius model linear fit for the calculation of the apparent activation energy of permeance in the Pebax®/ZIF-8/SWCNT-bp-SC for CO_2_ (A) and N_2_ (B). R2 fitting parameter is 0.97 and 0.94, respectively for CO_2_ and N_2_; Figure S3. CO_2_/N_2_ bound defined in GPU at 35 °C, adapted from the data published in Robeson [7]; Table S1. Gas permeation and selectivity results at 35 °C and 3 bar feed pressure.
