# Supplementary material for: Double-Layered Pebax® 3533/ZIF-8 Membranes with Single-Walled Carbon Nanotube Buckypapers as Support for Gas Separation
Source: Membranes (Basel). 2023 Jan 6;13(1):71. doi: 10.3390/membranes13010071 (PMC9860796; doi:10.3390/membranes13010071)
Supplement: Supplementary file 1 [file membranes-13-00071-s001.zip › membranes-2134874-supplementary.pdf]

## Supplementary information

# Double layered Pebax® 3533/ZIF-8 membranes with single-walled carbon nanotubes buckypaper as support for gas separation

Víctor Berned-Samatán,[1,2] Carlos Téllez[1,2] and Joaquín Coronas[1,2]*

[1]Instituto de Nanociencia y Materiales de Aragón (INMA), CSIC-Universidad de Zaragoza, Zaragoza 50018, Spain.

[2]Chemical and Environmental Engineering Department, Universidad de Zaragoza, Zaragoza 50018, Spain.

*Corresponding author: coronas@unizar.es

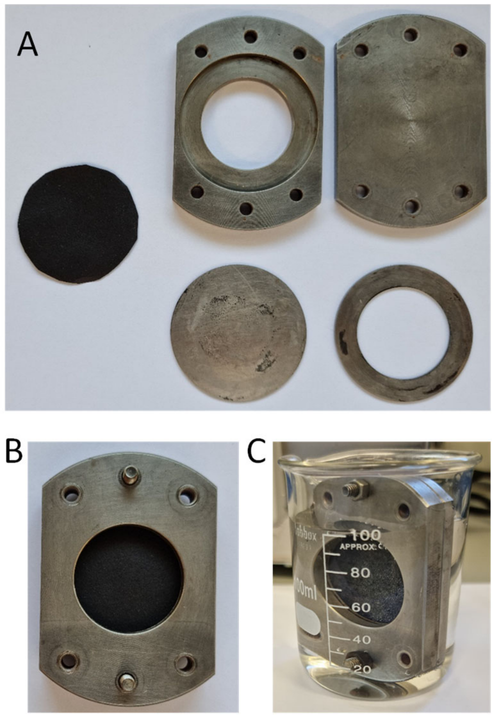

*Figure S1. Photographs of the experimental rig for the synthesis of the ZIF-8 layer on the SWCNT buckypaper (A). The buckypaper placed between the disc and the ring (B). ZIF-8 layer being synthesized on the buckypaper (C) at room temperature.*

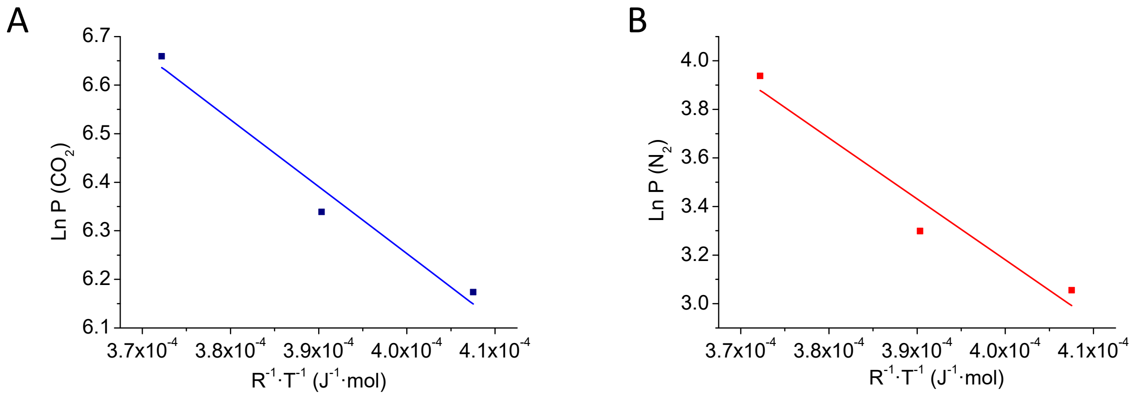


*Figure S2. Arrhenius model linear fit for the calculation of the apparent activation energy of permeance in the Pebax®/ZIF-8/SWCNT-bp SC for $CO_2$ (A) and $N_2$ (B). $R^2$ fitting parameter is 0.97 and 0.94, respectively for $CO_2$ and $N_2$.*

## Supplementary tables

*Table S1. Gas permeation and selectivity results at 35 °C and 3 bar feed pressure.*

| Membrane | $CO_2$ Permeance (GPU) | $CO_2/N_2$ Selectivity |
|---|---|---|
| **Dense Pebax® 3533** | 2.4 ± 1.8 | 21.0 ± 2.1 |
| **Pebax®/SWCNT-bp-PI** | 73 ± 9 | 14.7 ± 3.1 |
| **Pebax®/SWCNT-bp-SC** | 161 ± 12 | 18.3 ± 3.5 |
| **Pebax®/ZIF-8/SWCNT-bp-PI** | 115 ± 11 | 19.1 ± 2.2 |
| **Pebax®/ZIF-8/SWCNT-bp-SC** | 566 ± 23 | 20.9 ± 4.2 |

## Robeson upper bound adapted

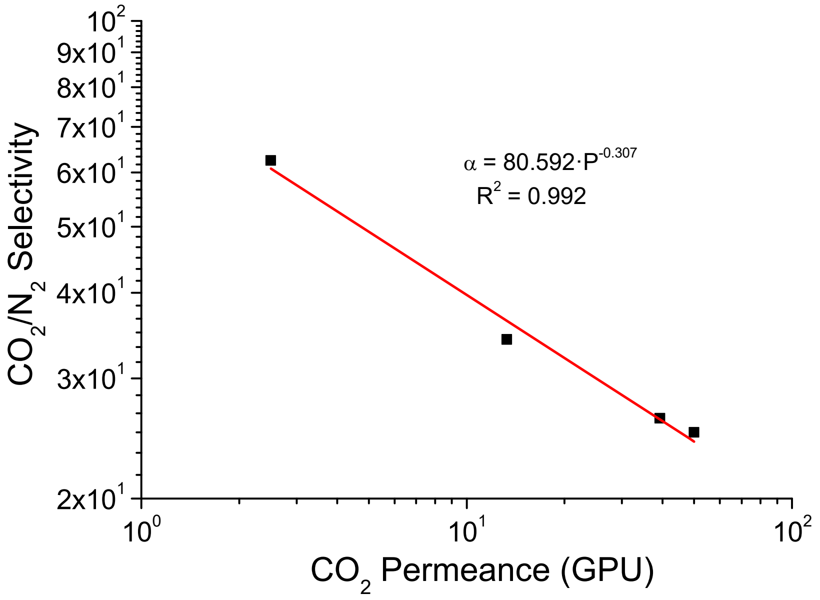


*Figure S3. $CO_2/N_2$ bound defined in GPU at 35 °C, adapted from the data published in Robeson .*[1]

### References


[1] L.M. Robeson, The upper bound revisited, J. Memb. Sci. 320 (2008) 390–400. https://doi.org/10.1016/j.memsci.2008.04.030.
